# Work-life conflict and cardiovascular health: 5-year follow-up of the Gutenberg Health Study

**DOI:** 10.1371/journal.pone.0251260

**Published:** 2021-05-07

**Authors:** Janice Hegewald, Karla Romero Starke, Susan Garthus-Niegel, Andreas Schulz, Matthias Nübling, Ute Latza, Sylvia Jankowiak, Falk Liebers, Karin Rossnagel, Merle Riechmann-Wolf, Stephan Letzel, Natalie Arnold, Manfred Beutel, Emilio Gianicolo, Norbert Pfeiffer, Karl Lackner, Thomas Münzel, Philipp Wild, Andreas Seidler

**Affiliations:** 1 Institute and Policlinic of Occupational and Social Medicine (IPAS), Faculty of Medicine Carl Gustav Carus, TU Dresden, Germany; 2 Institute of Sociology, Faculty of Behavioral and Social Sciences, TU Chemnitz, Chemnitz, Germany; 3 Department of Child Health and Development, Norwegian Institute of Public Health, Oslo, Norway; 4 University Medical Center of the Johannes Gutenberg University of Mainz, Mainz, Germany; 5 FFAW: The Freiburg Research Centre for Occupational Sciences, Freiburg, Germany; 6 Division Work and Health, Federal Institute for Occupational Safety and Health (BAuA), Berlin, Germany; 7 Institute for Teachers’ Health, University Medical Center of the Johannes Gutenberg University of Mainz, Mainz, Germany; 8 Institute of Occupational, Social, Environmental Medicine, University Medical Center of the Johannes Gutenberg University of Mainz, Mainz, Germany; 9 Center for Cardiology I, University Medical Center of the Johannes Gutenberg University of Mainz, Mainz, Germany; 10 Department of Medicine 2, Preventive Cardiology and Preventive Medicine, University Medical Center of the Johannes Gutenberg University of Mainz, Mainz, Germany; 11 Center for Translational Vascular Biology (CTVB), University Medical Center of the Johannes Gutenberg University of Mainz, Mainz, Germany; 12 Department of Psychosomatic Medicine and Psychotherapy, University Medical Center Mainz, Johannes Gutenberg University Mainz, Mainz, Germany; 13 Institute of Medical Biostatistics, Epidemiology and Informatics, University Medical Center of the Johannes Gutenberg University Mainz, Mainz, Germany; 14 Institute of Clinical Physiology, National Research Council, Lecce, Italy; 15 Department of Ophthalmology, University Medical Center Mainz, Johannes Gutenberg University Mainz, Mainz, Germany; 16 Institute for Clinical Chemistry and Laboratory Medicine, University Medical Center Mainz, Mainz, Germany; 17 DZHK (German Center for Cardiovascular Research), Partner Site Rhine-Main, Mainz, Germany; 18 Center of Thrombosis and Hemostasis (CTH), University Medical Center Mainz, Mainz, Germany; Medical University Innsbruck, AUSTRIA

## Abstract

**Introduction:**

Work-life conflicts (WLC) may impact health, but few studies prospectively consider the impact of WLC on objective outcomes such as cardiovascular disease. Using data from the Gutenberg Health Study (GHS), we examined if WLC at baseline was associated with an increased five-year incidence of cardiovascular events (myocardial infarct, stroke, atrial fibrillation, peripheral artery disease, coronary artery disease, chronic heart failure, sudden cardiac death). We also considered if WLC was associated with incident hypertension and arterial stiffness and if the effects of WLC on cardiovascular health differ for men and women.

**Methods:**

A working subsample of the 15,010 GHS cohort participants completed the Copenhagen Psychosocial Questionnaire, which included five "work-privacy conflict" questions at baseline and at the five-year follow-up. Relative risks for incident hypertension due to increased WLC at baseline (WLC scores exceeding 60 out of 100) were estimated with Poisson regression in the subgroup of participants without hypertension at baseline (n = 2426). Categories of WLC at baseline and follow-up were also used to examine the risk of hypertension due to chronic/recurrent WLC. In this subgroup, we also examined the association between WLC as a continuous score ranging from 0 to 100 with change to arterial stiffness after five years using linear regression. Hazard ratios were estimated for incident cardiovascular events in a larger subsample of participants without prevalent cardiovascular disease at baseline (n = 3698) using Cox regression. We used various multivariable regression models to adjust for sex, age, socioeconomic status, occupational, household, and cardiovascular risk factors.

**Results:**

We found no association between WLC and incident hypertension or increased arterial stiffness. The fully-adjusted relative risk for WLC >60 at baseline and hypertension was 0.93 (95% 0.74–1.17). The risk of hypertension due to chronic/recurrent WLC >60 was increased but not statistically significant (RR = 1.13, 95% CI 0.83–1.54). Overall, hazard ratios for incident cardiovascular events were also not increased. However, stratifying the results by sex resulted in a hazard ratio of 1.47 (95% CI 0.54–3.98) for incident cardiovascular disease among women in the fully adjusted model.

**Conclusions:**

Although our results were not statistically significant, they indicate that WLC is negatively impacting the cardiovascular health of women. While these results need to be confirmed with additional research and a longer follow-up, interventions to prevent WLC will promote health and could be especially beneficial for women.

## Introduction

Work-family conflict and work-life conflict are synonymous terms for the role conflict that occurs when occupational obligations are perceived to interfere with domestic or private life. Cross-sectional studies examining conflicts between work and family roles find these to be negatively associated with mental and self-reported physical health. [1–5]. However, prospective studies examining associations between WLC and health are still lacking.

Several population-based cross-sectional studies conducted in the USA found increasing levels of work-family conflicts to be associated with lower levels of mental health and self-reported physical health [1, 2, 5]. A survey conducted by Davis et al. [1] found that work-family conflict was associated with increased fatigue, poorer perceived physical health, and an increased number of reported health conditions. Minnotte et al. analyzed survey data from the National Study of the Changing Workforce and determined increased work-family conflict was associated with lower levels of mental health and poorer self-reported physical health [2]. A mediation analysis using the General Social Survey found that work-family conflict completely mediated the association between shift work and poorer self-reported general health, as well as the number of days with poor mental health or poor physical health in the last month [5]. Cross-sectional studies conducted in healthcare settings in Switzerland found work-family conflict was a strong predictor of burnout symptoms [3, 4]. Evidence from systematic reviews finds work-related psychosocial stress is associated with an increased risk of cardiovascular disease [6], but the mechanisms involved are not yet fully understood. One possibility is that psychosocial stress leads to unhealthy behavioral changes that indirectly increase the risk of cardiovascular diseases, such as poor dietary choices, smoking, over-consumption of alcohol, and reduced physical activity. However, Chandola et al. found psychosocial stress to also have a direct effect on cardiovascular disease [7]. Chronic exposure to stress can lead to allostatic overload resulting from the body’s reaction to stress, which includes activation of the sympathetic nervous system and the hypothalamic-pituitary-adrenal axis, chronic inflammation, and mitochondrial dysfunction [8, 9]. Over time, these stress responses can lead to endothelial damage and hypertension. The distress caused by chronic conflicts between private and work roles and its contribution to the allostatic load may also be negatively impacting cardiovascular health. Research examining the relationship between work-life conflict and cardiovascular health has shown some associations between role conflicts and poorer cardiovascular health [10, 11]. However, few prospective studies of work-life conflict and objectively measured physical health outcomes exist to date [12]. Prospective studies are essential to establishing causality; by ensuring that the exposure precedes the outcome, reverse causality can be prevented.

Shockley and Allen [13] prospectively examined the short-term effects of episodic work-family conflicts on blood pressure and heart rate in a sample of volunteers. This study had the participants record each episode of work-family conflict over ten days while monitoring heart rate and blood pressure four times a day. Using multilevel modeling analysis, it was determined if increases in blood pressure or heart rate were predicted by an earlier conflict episode. Conflict episodes caused a statistically significant short-term increase in heart rate (ϒ = 0.04, p <0.05) but did not increase systolic or diastolic blood pressure. However, the immediate effects of role conflict episodes on blood pressure were moderated by family-supportive supervision at work, with both systolic and diastolic blood pressure increasing among participants with less family-supportive supervision at work.

A prospective study of the Swedish Longitudinal Occupational Survey of Health (SLOSH) cohort found conflict arising from work interfering with family life increased women’s odds of being emotionally exhausted and men’s odds of problematic alcohol consumption after two years, even after adjusting for baseline levels of the respective health measure [14]. Frone et al. [10] found conflicts arising from family interfering with work increased the risk for incident hypertension and that work interfering family conflicts increased heavy alcohol consumption after four years.

The concept of work-family conflicts can also be expanded to encompass private-life roles in general. In addition to the role one has within the traditional family structure, private-life roles can also include all roles outside of paid employment, such as leisure, religious, and community roles [15]. Thus, we use the term work-life conflict (WLC) here to describe the perceived conflicts that can occur between one’s work and private (including familiar) roles, as this term is commonly used in the literature to describe how work-life and private-life domains interact.

We previously assessed WLC using baseline data from the population-based Gutenberg Health Study (GHS) in Mainz, Germany [16]. The results from this cross-sectional analysis identified working conditions, personal attributes, and lifestyle factors associated with increased WLC. We found that although a higher proportion of men experienced high to very high WLC (27.4%) compared to women (23.0%), women had a higher risk for WLC after adjusting for factors such as working part-time (prevalence ratio [PR] = 1.25; 95% confidence interval [CI] 1.08–1.44). The factors most strongly predictive of WLC also differed between men and women at baseline. While some predictors of WLC were the same for both sexes (age, socioeconomic status, negative affectivity, time spent on hobbies, working part-time, nightshift work), stepwise model selection additionally selected diabetes, depressive symptoms, being divorced or separated, working night shifts more than seven days per month, and working in management as predictors of WLC among women. In comparison, the model selection for men additionally selected smoking, pack-years, time spent caring for relatives, time spent on household errands, and working more than 40 hours per week as predictors of WLC. These results indicate that men and women experience WLC differently.

The aim of the current study is to investigate the impact of WLC on cardiovascular health using a prospective study design. In this study, we examine (i) if WLC measured at baseline is associated with an increased incidence of hypertension and cardiovascular events after five years, (ii) if recurrent WLC (indicated by WLC measured at baseline and at the five-year follow-up) is associated with hypertension, and (iii) if the impact of WLC on cardiovascular health differs between men and women.

## Methods

### Population

We examined the effects of WLC on a subsample of the GHS cohort. The GHS is a single-center population-based cohort study that recruited a random sample of 35- to 74-year-old residents living in the city of Mainz and the district of Mainz-Bingen starting in 2007. The cohort was established to evaluate factors associated with numerous health outcomes but initially focused on examining risk-factors for myocardial infarction and cardiovascular mortality (primary outcomes), as well as stroke, non-cardiovascular mortality, heart failure, atrial fibrillation, and diabetes mellitus (secondary outcomes) [17]. At baseline, a total of n = 15,010 participants were recruited. Individuals who were mentally or physically unable to visit the study center for the examinations or unable to sufficiently communicate in German were excluded. The recruitment efficacy at baseline (the proportion of participants among all persons randomly selected, including those who could not be contacted) was 55.5%, and the cooperation proportion or response (the proportion of participants among all persons contacted) was 70.0% [18]. Baseline assessments included an assessment of social, lifestyle, and occupational factors, as well as examinations of cardiovascular health and function conducted at University Medical Centre in Mainz, Germany in 2007–2012 [19]. The ethics committee of the Rhineland-Palatinate Medical Association (review number: 837.020.07(5555)) and the data protection officer of the University Medical Center of the Johannes Gutenberg-University Mainz reviewed the study. Written informed consent was obtained from all participants.

One particular advantage of the GHS study is the detailed retrospective assessment of study participants’ occupations [20] and the ongoing assessment of psychosocial working conditions with instruments, such as the Copenhagen Psychosocial Questionnaire (COPSOQ). Approximately half of the study participants in paid employment (n = 3927) were given the COPSOQ [21] at baseline. The COPSOQ is an instrument that assesses psychosocial working factors using various scales [22]. The standard German version of the COPSOQ also included a five-item "work-privacy conflict" scale [23, 24] described in the section "Work-Life Conflict (WLC)" below. Only the study participants who received the COPSOQ at baseline were considered in this study.

To analyze the effects of WLC on cardiovascular health prospectively, we also only considered participants without prevalent cardiovascular outcomes at baseline. Cardiovascular health was examined in two subsamples of participants. In the first subsample, we considered arterial hypertension and arterial stiffness as two outcomes that typically do not present with clinical symptoms. We excluded 1501 persons with prevalent hypertension at baseline from this subsample. In addition, 279 (11.5%) participants were lost to follow up, and 12 participants without a blood pressure measurement at follow-up were excluded. The five-year incidence of hypertension was considered in the remaining 2135 persons. Among this same subgroup of participants, the change in arterial stiffness after five years was considered in 1691 people with arterial stiffness measurements at both baseline and follow-up.

**Fig 1:**
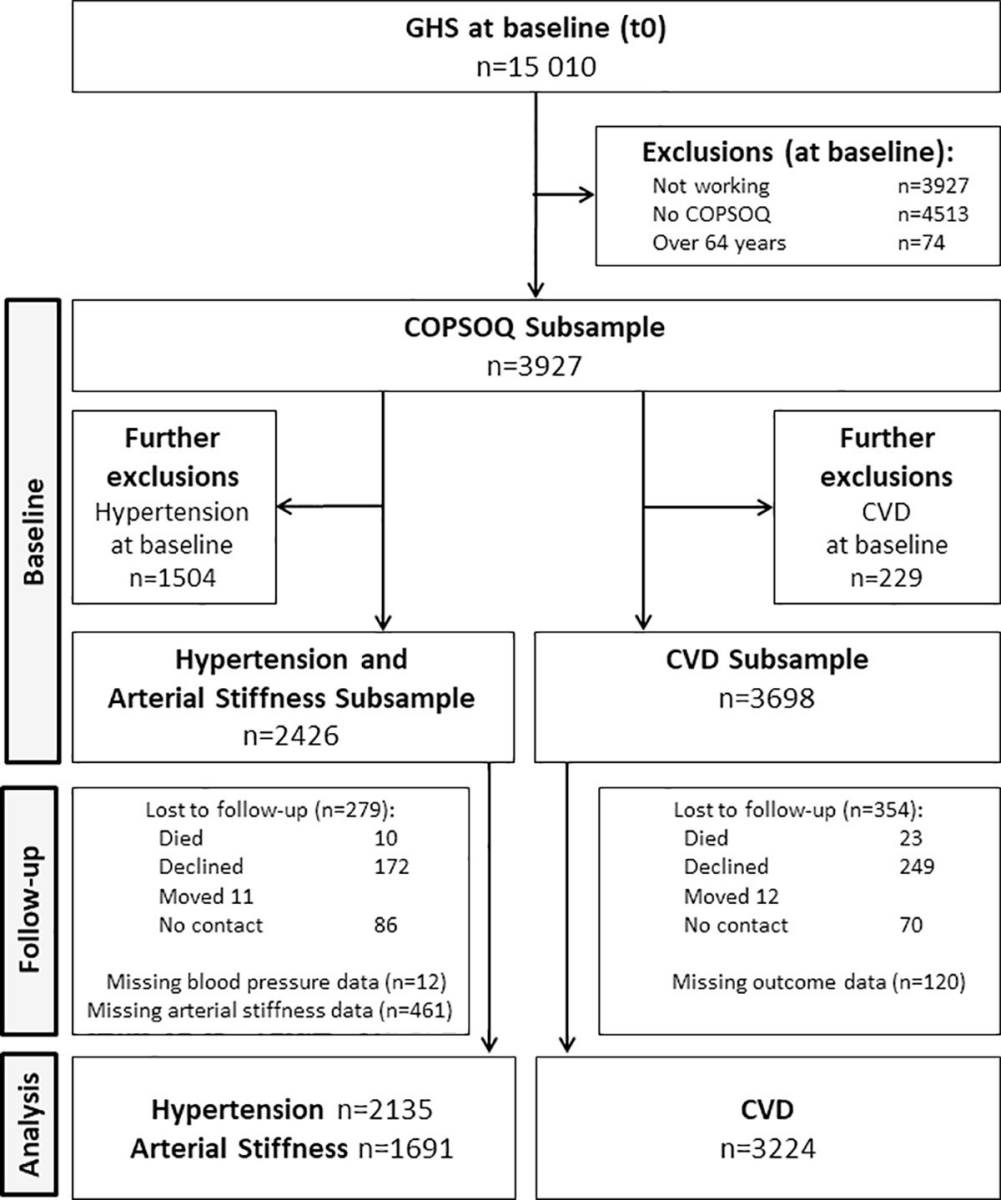
Study flow chart describing the selection of the hypertension/arterial stiffness and the CVD subsamples.

Cardiovascular disease (CVD) incidence was evaluated in a subsample of 3698 persons. This CVD subsample did not exclude participants with prevalent hypertension but excluded 229 people who reported having had a CVD (myocardial infarct (ICD-10: I21), cerebral infarction/ischemic stroke (ICD-10: I63), atrial fibrillation (ICD-10: I48), peripheral artery disease (ICD-10: I73.9), coronary artery disease (ICD-10: I25.10), chronic heart failure (ICD-10: I50, I11.0, I13.0, I13.2)) prior to or at baseline. In this subsample, 354 (9.6%) were lost to follow-up, and 120 (3.2%) people were missing information on CVD at the follow-up. A study flowchart depicting the subsamples of participants included in the analyses is shown in Fig 1.

### Work-Life Conflict (WLC)

We assessed WLC by rephrasing the Work-Family Conflict scale proposed by Netemeyer [25] to comprise all areas of personal life. WLC was assessed with the following five items:

1. The demands of my work interfere with my private and family life.
2. The amount of time my job takes up makes it difficult to fulfill family or private responsibilities.
3. Things I want to do at home do not get done because of the demands my job puts on me.
4. My job produces stress that makes it difficult to fulfill private or family duties.
5. Due to work-related duties, I have to make changes to my plans for private or family activities.

The items were rated with a five-item Likert scale [26] with strongly agree (5), agree (4), unsure/uncertain (3), disagree (2), or strongly disagree (1). Answers to the five items were combined and converted to a scale ranging from zero to 100, with higher values corresponding to increased levels of WLC. The association between continuous WLC values and change in arterial stiffness was analyzed using linear regression. We also analyzed WLC as a binary variable and considered WLC scores of >60 to indicate the presence of WLC. This cut-point was chosen for our baseline analysis [16] because it corresponds roughly with the two Likert-scale categories indicating high to very high WLC. The binary baseline WLC values were used to analyze the risks of incident hypertension and CVD. We also created a categorical variable describing exposure to WLC over time using binary WLC variables at baseline and follow-up. This categorical WLC was used to analyze the risk of incident hypertension due to ongoing or recurrent WLC and comprised the following levels: no increased WLC at either assessment time (reference), increased WLC only at baseline, WLC only at follow-up, and increased WLC scores at both baseline and at the five-year follow-up.

### Cardiovascular outcomes

Hypertension (ICD-10: I10) was defined as a mean systolic blood pressure of ≥140 mmHg or mean diastolic blood pressure of ≥90 mmHg in the 2nd and 3rd standardized measurement after 8 and 11 minutes of rest, or self-reported use of antihypertensive medications. Arterial stiffness was measured at both baseline and follow-up using digital photoplethysmography of the ring finger using a Pulse Trace PCA2 device (Micro Medical Limited/Carefusion). The arterial stiffness index was calculated as the height (in meters) divided by the difference between early systolic and second diastolic peak (in seconds) [27]. Change in arterial stiffness index occurring since baseline was evaluated as a continuous variable.

The risk for incident cardiovascular diseases was evaluated by considering acute myocardial infarct (ICD-10: I21), cerebral infarction/ischemic stroke (ICD-10: I63), atrial fibrillation (ICD-10: I48), peripheral artery disease (ICD-10: I73.9), coronary artery disease (ICD-10: I25.10), chronic heart failure (ICD-10: I50, I11.0, I13.0, I13.2), or confirmed sudden cardiac death (ICD-10: I46) occurring during the follow-up period. An outcome committee made up of medical experts reviewed hospital records and death certificates together to confirm all cardiovascular events.

### Potential confounders

Several adjustment sets (potential confounders) for the multivariable regression models were selected with Directed Acyclic Graphs (DAGs) [28]. The basic adjustment set (Model 1) included sex, age (continuous), and socioeconomic status (continuous). Socioeconomic status (SES) was measured using the score described by Lampert [29], which summarizes education level, occupational position, and household income in a score ranging from 3 (poorer status) to 21 (higher status).

In a subsequent model (Model 2), we additionally adjusted for occupational factors that could contribute to WLC and probably affect cardiovascular health, thereby causing confounding: working in a management position, night shift (working hours between 11 p.m. and 5 a.m.), and working hours per week. In a further model (Model 3), we also adjusted for private-life factors that could cause confounding by impacting the risks for WLC and cardiovascular health. These private-life factors included living with a partner, the number of children under the age of 18, time spent caring for children (hours per week), time spent caring for relatives (hours per week), time spent taking care of the household (hours per week), time spent on hobbies and sport (hours per week). As a sensitivity analysis (Model 4), we also adjusted for potential intermediate factors that could have been influenced by WLC (i.e., smoking status, alcohol abuse (female alcohol intake >40g/day; male alcohol intake >60g/day), and waist to height ratio (WHtR)). This sensitivity analysis provided an estimate of the direct effect of WLC on cardiovascular outcomes.

### Statistical analysis

Relative risks (RR) and 95% confidence intervals (CI) for incident hypertension were estimated using Poisson regression models with a robust variance estimation. For these models, we considered WLC at baseline using the cutoff of WLC >60 (versus WLC scores ≤60 as the reference). Risk of hypertension due to WLC exposure at both baseline and follow-up was also considered using the categories of WLC described in the section "Work-Life Conflict (WLC)".

Change in arterial stiffness since baseline was modeled with linear regression models. Here we considered WLC as a continuous variable and reported the change in arterial stiffness per 10-point increase in WLC scores (β). An interaction term for WLC and sex was also included in the arterial stiffness models to examine if the effect of WLC on arterial stiffness differed between men and women.

Hazard ratios (HR) for CVD were estimated using Cox regression models for competing risks, where competing risk events were non-CVD deaths and the time scale was the time in study (years). For these models, only WLC at baseline (binomial WLC > 60) was considered.

The adjustment sets mentioned above were used to adjust for confounding. We also stratified all analyses by sex to examine if the exposure-risk relationship differed between men and women. To prevent selection bias due to missing data, medians and modes were used to impute missing values of adjustment factors included in the multivariable regression models. The statistical analysis was conducted using R-project version 3.3.1 [30].

## Results

A total of 3698 study participants with WLC scores at baseline were considered in the main analysis of incident CVD. Due to the high prevalence of hypertension at baseline (38.2%, n = 1501), the five-year incidence of hypertension was considered in a smaller subsample (n = 2426). The characteristics of the sample populations at baseline are shown in Table 1.

**Table 1:** Population characteristics at baseline.

	**CVD subsample**			**Hypertension/Arterial Stiffness subsample**		
	Total (N = 3698)	WLC ≤60 (N = 2928)	WLC >60 (N = 770)	Total (N = 2426)	WLC ≤60 (N = 1900)	WLC >60 (N = 526)
**Sex** Women	44.7% (1653)	46.2% (1352)	39.1% (301)	49.0% (1189)	50.7% (964)	42.8% (225)
Men	55.3% (2045)	53.8% (1576)	60.9% (469)	51.1% (1237)	49.3% (936)	47.2% (301)
**Age** in years, mean (SD)	48.0 (7.5)	48.2 (7.5)	47.1 (7.3)	46.5 (7.3)	46.7 (7.3)	45.9 (7.2)
**Socioeconomic Status (SES)** (range 3 to 21), mean (SD)	14.31 (4.14)	14.00 (4.11)	15.49 (4.03)	14.47 (4.07)	14.17 (4.03)	15.59 (4.02)
**Part-time Employment**	21.7% (804)	24.6% (721)	10.8% (83)	23.7% (575)	26.9% (511)	12.2% (64)
**Management Position**	16.2% (598)	14.2% (417)	23.5% (181)	14.3% (348)	12.6% (239)	20.7% (109)
**Night shift work**	13.5% (501)	11.5% (338)	21.2% (163)	13.6% (331)	12.0% (228)	19.6% (103)
**Work per week [hours]**	37.43 (11.62)	36.13 (10.97)	42.37 (12.67)	36.80 (11.62)	35.50 (11.04)	41.50 (12.43)
**Living in Partnership**	81.1% (2998)	81.1% (2376)	80.8% (622)	80.4% (1951)	80.3% (1526)	80.8% (425)
**Children (yes)**	71.9% (2659)	72.2% (2115)	70.6% (544)	70.4% (1709)	70.9% (1347)	68.8% (362)
**Number of children,** mean (SD)	1.36 (1.10)	1.36 (1.09)	1.37 (1.13)	1.35 (1.10)	1.35 (1.10)	1.33 (1.12)
**Number of children <18 years,** mean (SD)^**a**^	0.13 (0.51)	0.12 (0.50)	0.15 (0.53)	0.15 (0.55)	0.14 (0.55)	0.17 (0.56)
**Time spent on caring for children [hours/week]**^**b**^	2.07 (2.58)	2.13 (2.56)	1.90 (2.64)	2.24 (2.54)	2.22 (2.39)	2.30 (2.97)
**Time spent on caring for relatives [hours/week]**^**b**^	0.11 (0.52)	0.10 (0.51)	0.14 (0.54)	0.10 (0.47)	0.09 (0.44)	0.15 (0.57)
**Time spent on household [hours/week]**^**c**^	1.80 (1.00/3.00)	2.00 (1.10/3.00)	1.50 (1.00/2.50)	1.90 (1.00/3.00)	2.00 (1.20/3.00)	1.50 (1.00/2.50)
**Time spent on hobbies/sport [hours/week]**^**c**^	1.00 (0.50/2.00)	1.00 (0.50/2.00)	1.00 (0.33/2.00)	1.00 (0.50/2.00)	1.00 (0.50/2.00)	1.00 (0.45/2.00)
**Smokers**	23.2% (858)	23.6% (690)	21.8% (168)	25.5% (618)	26.4% (502)	22.1% (116)
**Pack-years**^**c**^	0.17 (0/3.51)	0.21 (0/3.69)	0.06 (0/2.80)	0.14 (0/3.48)	0.16 (0/3.62)	0.04 (0/2.42)
**Alcohol per day [g]** ^**c**^	5.03 (0/17.44)	5.03 (0/16.47)	6.29 (0/18.86)	5.03 (0/14.94)	4.55 (0/13.49)	6.29 (0/18.65)
**Alcohol abuse**^d^	2.7% (100)	2.8% (81)	2.5% (19)	2.1% (52)	2.1% (40)	2.3% (12)
**WHtR**	0.54 (0.07)	0.53 (0.07)	0.54 (0.07)	0.52 (0.07)	0.52 (0.07)	0.52 (0.07)
**Baseline Hypertension**	37.0% (1367)	37.9% (1109)	33.6% (258)	0	0	0
**SBP [mmHg]**	127.4 (15.2)	127.8 (15.5)	125.9 (13.9)	120.1 (9.9)	120.1 (10.1)	120.1 (9.2)
**DBP [mmHg]**	82.8 (9.4)	82.9 (9.4)	82.6 (9.2)	78.4 (6.5)	78.3 (6.5)	78.8 (6.5)
**Antihypertensive medications**^**e**^ (self reported)†	25.8% (839 of 3249)	26.5% (684 of 2578)	23.1% (155 of 671)	6.1% (131 of 2136)	6.4% (107 of 1682)	5.3% (24 of 454)
**Stiffness Index [m/s]**	7.19 (2.06)	7.20 (2.07)	7.16 (2.01)	6.86 (1.93)	6.86 (1.93)	6.89 (1.92)

SBP systolic blood pressure; DBP diastolic blood pressure; WHtR waist-to-height ratio.

^a^Describes average only among persons with children.

^b^Time spent caretaking averaged only for households with children or with at least one other person living in the household, respectively.

^c^median (quartile 1/ quartile 3) because data were skewed.

^d^alcohol abuse: female alcohol intake >40g/day and male alcohol intake >60g/day.

^e^The number of persons self-reporting the use of antihypertensive medication differed from the total N due to missing values.

At baseline, 20.8% (n = 770) of the participants in the CVD subsample and 21.7% (n = 526) of the participants in the hypertension subsample had WLC scores >60. The characteristics of the subsamples were similar, but excluding persons with prevalent hypertension lowered the average age of the incident hypertension subsample by two years. In both subsamples, fewer participants experiencing increased WLC at baseline worked part-time, while more individuals with WLC worked in a management position or worked night shifts. Smoking was less frequent, and the average pack-years smoked were lower among persons with increased WLC, while the reported average alcohol consumed per day (grams) was slightly higher. Family characteristics at baseline were similar for both subsamples and did not differ according to WLC levels. The proportions of participants living with a partner or having children did not differ much across WLC categories, but persons with increased WLC spent about half an hour less per week caring for their household. The distribution of incident hypertension and CVD events according to WLC scores is shown in the supporting information (S1 and S2 Tables). The median follow-up time was 5.0 years for both subgroups, and the average follow-up was 4.4 years for the hypertension/arterial stiffness subsample and 4.8 years for the CVD subsample.

### Incident hypertension

Increased WLC at baseline did not increase the risk for incident hypertension after five years (Table 2). The relative risks (RR) for the entire population and men were consistently below one, regardless of the adjustment set. The RR for women indicated a small (not statistically significant) increase in risk that disappeared in the fully adjusted model (including potential intermediate factors).

**Table 2:** Risk ratios (RR) and 95% confidence intervals (CI) for WLC and incident hypertension.

		**Cases/Participants**	**RR**^a^ **(95% CI)**	**RR**^b^ **(95% CI)**	**RR**^c^ **(95% CI)**	**RR**^d^ **(95% CI)**
**All (n = 2135)**	WLC ≤60	314/1680	1 (reference)	1 (reference)	1 (reference)	1 (reference)
	WLC >60	77/455	0.94 (0.75–1.18)	0.94 (0.75–1.18)	0.95 (0.75–1.20)	0.93 (0.74–1.17)
**Women**	WLC ≤60	121/849	1 (reference)	1 (reference)	1 (reference)	1 (reference)
	WLC >60	28/194	1.06 (0.73–1.54)	1.07 (0.73–1.54)	1.08 (0.74–1.58)	0.99 (0.74–1.58)
**Men**	WLC ≤60	193/831	1 (reference)	1 (reference)	1 (reference)	1 (reference)
	WLC >60	49/261	0.87 (0.66–1.16)	0.88 (0.66–1.17)	0.89 (0.67–1.18)	0.89 (0.68–1.18)

RR relative risk; CI confidence interval; WLC work-life conflict.

^a^Model 1: sex (excluded from stratified models), age, SES, working hours per week.

^b^Model 2: sex (excluded from stratified models), age, SES, working hours, management, night shift.

^c^Model 3: sex (excluded from stratified models), age, SES, working hours, management, night shift, living with a partner, time spent caring for children, time spent caring for relatives, time spent on household, time spent on hobbies/sport.

^d^Model 4: sex (excluded from stratified models), age, SES, working hours, management, night shift, living with a partner, time spent caring for children, time spent caring for relatives, time spent on household, time spent on hobbies/sport, smoking, alcohol abuse, WHtR (per SD).

In the total population, risks for incident hypertension increased among persons with WLC scores of >60 at both baseline and follow-up, which we considered indicative of possibly chronic or, at the least, recurrent WLC (Table 3). When women were considered separately, risks for incident hypertension were highest for women experiencing increased WLC at follow-up only. Adjusting for occupational and private conditions at baseline did not change the risk estimates much, but additionally, adjusting for smoking, alcohol abuse, and WHtR resulted in reduced risk estimates. In comparison, risk estimates for incident hypertension increased only among men experiencing WLC at both assessment points. As with the total sample, the sex-stratified results were also not statistically significant.

**Table 3:** Risk ratios (RR) and 95% confidence intervals (CI) for recurrent WLC and incident hypertension.

	**Cases/Participants**	**RR**^a^ **(95% CI)**	**RR**^b^ **(95% CI)**	**RR**^c^ **(95% CI)**	**RR**^d^ **(95% CI)**
**All (n = 1854)**					
No WLC at both times	234/1315	1 (reference)	1 (reference)	1 (reference)	1 (reference)
WLC >60 at baseline	29/202	0.86 (0.60–1.22)	0.87 (0.61–1.25)	0.88 (0.62–1.26)	0.87 (0.60–1.24)
WLC >60 at follow-up (incident)	24/139	1.05 (0.72–1.54)	1.07 (0.73–1.57)	1.06 (0.72–1.55)	0.97 (0.67–1.41)
Chronic/recurrent WLC >60	38/198	1.12 (0.83–1.53)	1.14 (0.84–1.55)	1.15 (0.84–1.57)	1.13 (0.83–1.54)
**Women (n = 900)**					
No WLC at both times	86/662	1 (reference)	1 (reference)	1 (reference)	1 (reference)
WLC >60 at baseline	13/96	1.06 (0.62–1.80)	1.05 (0.61–1.82)	1.08 (0.63–1.85)	0.94 (0.54–1.61)
WLC >60 at follow-up (incident)	10/70	1.18 (0.65–2.18)	1.19 (0.65–2.19)	1.21 (0.65–2.24)	1.14 (0.63–2.05)
Chronic/recurrent WLC >60	9/72	1.05 (0.55–2.00)	1.10 (0.58–2.12)	1.10 (0.57–2.13)	0.99 (0.50–1.94)
**Men (n = 954)**					
No WLC at both times	148/653	1 (reference)	1 (reference)	1 (reference)	1 (reference)
WLC >60 at baseline	16/106	0.74 (0.46–1.19)	0.75 (0.47–1.20)	0.75 (0.47–1.21)	0.77 (0.48–1.25)
WLC >60 at follow-up (incident)	14/69	0.98 (0.60–1.59)	0.99 (0.60–1.61)	0.97 (0.59–1.59)	0.91 (0.56–1.48)
Chronic/recurrent WLC >60	29/126	1.14 (0.80–1.62)	1.15 (0.81–1.64)	1.16 (0.82–1.65)	1.18 (0.83–1.66)

RR relative risk; CI confidence interval; WLC work life conflict

^a^Model 1: sex (excluded from stratified models), age, SES, working hours per week.

^b^Model 2: sex (excluded from stratified models), age, SES, working hours, management, night shift.

^c^Model 3: sex (excluded from stratified models), age, SES, working hours, management, night shift, living with partner, number of children under 18 years, time spent caring for children, time spent caring for relatives, time spent on household, time spent on hobbies/sport.

^d^Model 4: sex (excluded from stratified models), age, SES, working hours, management, night shift, living with a partner, time spent caring for children, time spent caring for relatives, time spent on household, time spent on hobbies/sport, smoking, alcohol abuse, WHtR (per SD).

### Arterial stiffness

The analysis of changes to arterial stiffness after 5-years showed no statistically significant association between WLC and change to arterial stiffness (Table 4). There was also no clear indication of sex interacting with WLC to cause endothelial changes. The age, sex, and SES-adjusted regression models indicated a positive association between the arterial stiffness index per 10-point increase in baseline WLC that was higher among women, but not none of the results were statistically significant. The interaction term between sex and WLC also failed to reach statistical significance.

**Table 4:** Linear regression models of change in arterial stiffness index since baseline.

Δ Stiffness Index (m/s)	Total (n = 1691)		Men (n = 988)	Women (n = 703)
	β^a^ (95% CI)	β ^a^ (95% CI)	β ^a^ (95% CI)	β ^a^ (95% CI)
WLC score (per 10)	0.03 (-0.02 to 0.07)	0.01 (-0.05 to 0.08)	0.02 (-0.5 to 0.09)	0.04 (-0.2 to 0.10)
Sex * WLC score	-	0.03 (-0.07 to 0.12)	-	-

^a^adjusted for sex (excluded from stratified models), age, SES.

### Incident CVD

Altogether, 109 incident cardiovascular events and 22 competing events (non-CVD deaths) occurred among the entire COPSOQ subsample during the five-year follow-up. The Cox regression models of the entire population sample estimated a sex, age and SES-adjusted HR of 1.09 (95% CI 0.68–1.75). Adjusting for occupational, household, and cardiovascular risk factors did little to change the risk estimates of the whole population. The stratified models indicated that men with increased WLC did not have an increased five-year risk for CVD, and the estimated HR was 1.00 for the fully adjusted model. In contrast, we observed increased but also not statistically significant risk estimates for women. The sex, age, and SES-adjusted HR for women was 1.27 (95% CI 0.49–3.28), and this increased to 1.56 (95% CI 0.57–4.24) with adjustment for occupational and household factors. Further adjustment (fully adjusted model) for CVD risk factors that could be intermediate factors between WLC and incident CVD events mitigated the estimated HR some (Table 5).

**Table 5:** Hazard ratios (HR) and 95% confidence intervals (CI) for incident CVD and WLC scores exceeding 60 (of 100) from competing event analysis (competing event = non-CVD deaths).

	**Hazard Ratio (HR)** ^a^ **(95% CI)**	**HR** ^b^ **(95% CI)**	**HR** ^c^ **(95% CI)**	**HR** ^d^ **(95% CI)**
**Total (n = 3596)** 102 events, 22 competing events	1.09 (0.68–1.75)	1.10 (0.67–1.81)	1.09 (0.66–1.81)	1.08 (0.65–1.80)
**Women (n = 1607)** 25 events, 7 competing events	1.27 (0.49–3.28)	1.36 (0.49–3.78)	1.56 (0.57–4.24)	1.47 (0.54–3.98)
**Men (n = 1989**) 77 events, 15 competing events	1.03 (0.59–1.77)	1.03 (0.59–1.82)	1.02 (0.57–1.82)	1.00 (0.56–1.80)

^a^Model 1: sex (excluded from stratified models), age, SES, working hours per week.

^b^Model 2:sex (excluded from stratified models), age, SES, working hours, management, night shift.

^c^Model 3: sex (excluded from stratified models), age, SES, working hours, management, night shift, living with partner, number of children under 18 years, time spent caring for children, time spent caring for relatives, time spent on household, time spent on hobbies/sport.

^d^Model 4: sex (excluded from stratified models), age, SES, working hours, management, night shift, living with partner, number of children under 18 years, time spent caring for children, time spent caring for relatives, time spent on household, time spent on hobbies/sport, smoking, alcohol abuse, WHtR (per SD).

## Discussion

We found divergent strengths of associations for men and women. Most notably, we detected tentatively increased risks (not statistically significant) of incident CVD events only for women. The HR for women remained increased at 1.47 (95% CI 0.54–3.98) after adjustment for occupational, household, and CVD risk factors, while the same adjusted model for men resulted in an HR of 1.00 (95% CI 0.56–1.80). However, none of the results reached statistical significance. Although there was no clear indication of any association between incident hypertension and arterial stiffness with WLC, the risk of incident hypertension and changes in arterial stiffness were also minimally increased for women but not for men.

While the results were not statistically significant, the observed increased risk estimates for women suggest a difference in health effects due to WLC. If this is true, it contradicts the summary of various findings cited by Frone [15], who concluded that the evidence from cross-sectional and longitudinal studies up to 2003 gave no indication of gender modifying the health effects of work-family conflict or family-work conflicts. However, the more recent prospective Swedish Longitudinal Occupational Survey of Health study did find differences between how WLC affects the health of men and women, with WLC increasing the odds of poor self-rated health among women and problem drinking among men [14].

If WLC experienced at baseline does have any impact on women’s cardiovascular health but not on men’s, it is unclear what would cause such a difference. One possibility is that the allostatic load resulting from WLC [31] was more pronounced for women. Despite increasing equality between men and women regarding workforce participation and working time, the corresponding distribution of domestic tasks between heterosexual partners that follows may be slowed by long-standing social expectations, and married women in paid employment continue to spend a disproportionate amount of time on household activities [32]. Knežević [33] found while men and women attribute similar salience or perceived importance to their spousal, familiar, and parental roles, women still spent more time and energy on these roles compared to men despite similar expenditures of time and energy on work-related roles. In contrast, Knežević et al. [33] found men spent more time and energy on leisure roles. The baseline analysis of the Brazilian Longitudinal Study of Adult Health (ELSA-Brasil) also found that strain-based work interfering with family and family interfering with work was associated with lower cardiovascular health scores for lifestyle factors (i.e., diet, physical activity, smoking, and body mass index) among women but not men [34].

Despite using a prospective analysis to examine health effects, some reverse causality may also explain some of the cardiovascular effects of WLC we observed and possibly some of the differential effects of WLC on women’s health. Poor health can result in decreased working ability and absences from work, which may cause these employees to "drift" to employment with less favorable conditions, and this could result in increased WLC ("drift hypothesis") [35]. Our own cross-sectional baseline analysis found prevalent depression was associated with WLC only among women [16]. However, it is unclear if depressive symptoms at baseline led to the increased WLC at baseline or if WLC was a contributing cause of depression. Nevertheless, this association between depression and WLC among women at baseline could also be associated with the increase in the five-year CVD incidence among women.

One asset of this research was that it was conducted with data from a large prospective population-based study with an extensive occupational history. Another strength of this research was that incident CVD events were assessed with medical records and confirmed by a committee of experts. While this outcome assessment was not actively blinded to job strain factors, the outcome assessors were not aware of WLC levels. The analysis of incident CVD events also reduced bias due to loss-to-follow-up, since the obtainment of hospital and vital records was conducted without participants’ needing to physically attend the follow-up assessment. In addition, since only the effect of baseline WLC on incident CVD risk was considered, these analyses are unlikely to be impacted by healthy worker bias.

### Study limitations

Although the cohort was quite large, the analyses may have still lacked statistical power to find the increased effect estimates observed among women. Few incident events were observed in the first five years among women, and the confidence intervals of the estimated events were wide. This problem should be alleviated with a longer follow-up period, as the number of CVD events observed over time should increase the statistical power of future analyses. Since we could only include participants with data available at both assessment times in the analyses of incident hypertension and arterial stiffness, these analyses may have been more prone to loss to follow-up and healthy worker bias. Especially the analysis of recurrent WLC may have been impacted by healthy worker bias, because 281 participants (13%) who were at follow-up but missing a WLC score at follow-up were excluded from these models. Only people who were still working would have completed the WLC assessment at the follow-up, and participants with health problems or with WLC may have been more likely to retire or quit working before the five-year follow-up.

While this research focuses on the directional role conflict that occurs specifically between work and family life (work interfering with family), role conflicts can also arise from family roles interfering with work (family interfering with work) [36]. Unfortunately, we could not estimate the effect of private-life roles interfering with work on cardiovascular health. Research shows that these reciprocal forms of conflicts, WLC and Life-Work Conflict, are distinct but related constructs [37]. Conflicts arising from private-life factors may have different effects on health. For example, Bergs et al. [37] found conflicts arising from private life had an effect on later depressive complaints, while conflicts arising from work did not. Also, we have no information on the effects of work facilitating private-life roles ("work-life balance"), which is also considered a distinct dimension of the interaction between work and private life [15].

Our results suggest that measures to reduce WLC could be useful to promote cardiovascular health, especially among women. A recent cross-sectional study of female nurses on rapidly rotating shift schedules found women on counterclockwise (backwards) rotation were more likely to report poorer work-life balance than women on a clockwise (forwards) rotation [38], suggesting that improved shift scheduling can already alleviate WLC. A systematic review of work-family intervention studies found that implementing alternative work arrangements, such as self-scheduling of shift work and "Family Supportive Supervisor Behavior Training" may reduce work-family conflict [39]. There is also some evidence that flexible working conditions [40] and support policies may have positive effects on health.

## Conclusions

We found tentative indications that experiencing increased WLC may be negatively impacting the cardiovascular health of women. On the other hand, we found no increased CVD risk for men during the five-year follow-up. While these results were not statistically significant and need to be confirmed with additional research, interventions to prevent WLC could promote health and be especially beneficial for women. However, changes in what is considered "normative" domestic roles and increasing egalitarianism with respect to household tasks may one day cause a redistribution of risks. Also, workplace changes, such as increasing digitalization, may increase work flexibility but could also be making work more intrusive so that the boundaries between work and private time are less clear. Continuing research on WLC is needed to determine how these societal and workplace changes will influence the prevalence of WLC and its impact on health.

## Supporting information

S1 Table Hypertension incidence according to WLC at baseline. (DOCX)

S2 Table CVD incidence according to WLC at baseline. (DOCX)
