# Correction: Work-life conflict and cardiovascular health: 5-year follow-up of the Gutenberg Health Study

**DOI:** 10.1371/journal.pone.0258075

**Published:** 2021-09-27

**Authors:** Janice Hegewald, Karla Romero Starke, Susan Garthus-Niegel, Andreas Schulz, Matthias Nübling, Ute Latza, Sylvia Jankowiak, Falk Liebers, Karin Rossnagel, Merle Riechmann-Wolf, Stephan Letzel, Natalie Arnold, Manfred Beutel, Emilio Gianicolo, Norbert Pfeiffer, Karl Lackner, Thomas Münzel, Philipp Wild, Andreas Seidler

Fig 1 is incorrect. The authors have provided a corrected version here.

**Fig 1:**
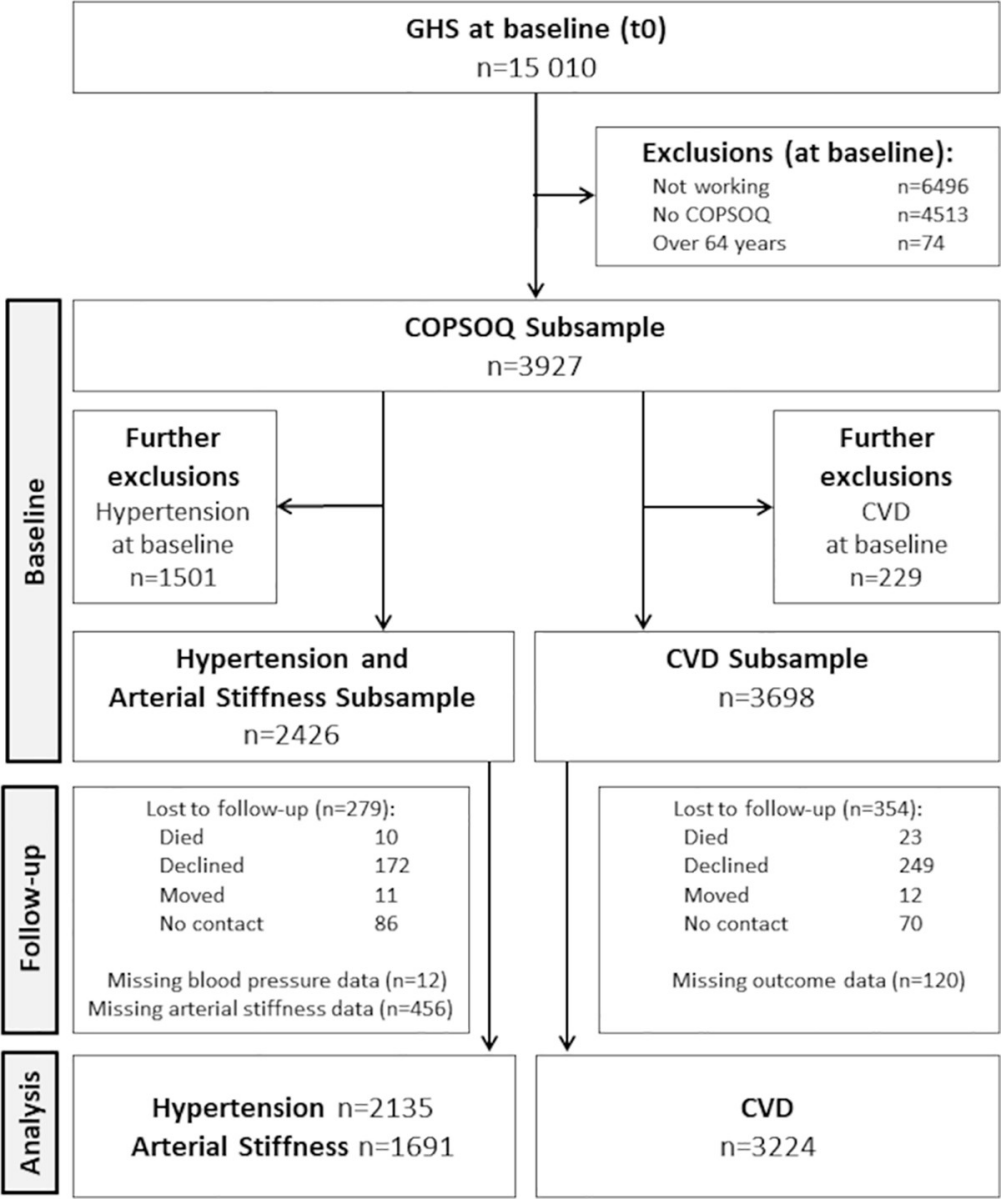
Study flow chart describing the selection of the hypertension/arterial stiffness and the CVD subsamples.
