# Supplementary material for: Work-life conflict and cardiovascular health: 5-year follow-up of the Gutenberg Health Study
Source: PLoS One. 2021 May 7;16(5):e0251260. doi: 10.1371/journal.pone.0251260 (PMC8104925; doi:10.1371/journal.pone.0251260)
Supplement: S1 Table — (DOCX) [file pone.0251260.s001.docx]

**S1 Table. Hypertension incidence according to WLC at baseline**

|  | Preclinical subsample | | | |
| --- | --- | --- | --- | --- |
|  | **Total†**  **(N=2135)** | **Incident hypertension**  **(N=391)** | **Men with incident hypertension**  **(N=242)** | **Women with incident hypertension**  **(N=149)** |
| **Baseline WLC Score, mean ± SD** | 37.57±26.95 | 36.36±26.77 | 36.82±26.94 | 35.60±26.57 |
|  |  |  |  |  |
| **Baseline WLC, n (%)** |  |  |  |  |
| 0-19 | 596 (27.9) | 107 (27.4) | 62 (25.6) | 45 (30.2) |
| 20-39 | 546 (25.6) | 113 (28.9) | 78 (32.2) | 35 (23.5) |
| 40-59 | 438 (20.5) | 82 (21.0) | 47 (19.4) | 35 (23.5) |
| 60-79 | 391 (18.3) | 59 (15.1) | 34 (14.0) | 25 (16.8) |
| 80-100 | 164 (7.7) | 30 (7.7) | 21 (8.7) | 9 (6.0) |
|  |  |  |  |  |
| **Follow-Up WLC Score, mean ± SD** | 33.99±26.32 | 34.35±26.30 | 35.85±26.16 | 31.74±26.45 |
|  |  |  |  |  |
| **Follow-Up WLC, n (%)** |  |  |  |  |
| 0-19 | 275 (28.8) | 105 (32.3) | 63 (30.4) | 42 (35.6) |
| 20-39 | 255 (26.7) | 86 (26.5) | 53 (25.6) | 33 (28.0) |
| 40-59 | 191 (20.0) | 59 (18.2) | 41 (19.8) | 18 (15.3) |
| 60-79 | 171 (17.9) | 57 (17.5) | 39 (18.8) | 18 (15.3) |
| 80-100 | 62 (6.5) | 18 (5.5) | 11 (5.3) | 7 (5.9) |
|  |  |  |  |  |
| **WLC over time** |  |  |  |  |
| No WLC at both times | 1315 (61.6) | 234 (59.8) | 148 (61.2) | 86 (57.7) |
| WLC >60 at baseline | 202 (9.5) | 29 (7.4) | 16 (6.6) | 13 (8.7) |
| WLC >60 at follow-up (incident) | 139 (6.5) | 24 (6.1) | 14 (5.8) | 10 (6.7) |
| Chronic/recurrent WLC >60 | 198 (9.3) | 38 (9.7) | 29 (12.0) | 9 (6.0) |
| no WLC at baseline, follow-up missing | 226 (10.6) | 56 (14.3) | 31 (12.8) | 25 (16.8) |
| WLC >60 at baseline, follow-up missing | 55 (2.6) | 10 (2.6) | 4 (1.7) | 6 (4.0) |

**†** Of the study sample considered at baseline (n=2426), information on hypertension at the follow-up was missing 12 study participants and 279 were lost to follow-up.
