# Supplementary material for: Work-life conflict and cardiovascular health: 5-year follow-up of the Gutenberg Health Study
Source: PLoS One. 2021 May 7;16(5):e0251260. doi: 10.1371/journal.pone.0251260 (PMC8104925; doi:10.1371/journal.pone.0251260)
Supplement: S2 Table — (DOCX) [file pone.0251260.s002.docx]

**S2 Table. CVD incidence according to WLC at baseline**

|  | CVD subsample | | | |
| --- | --- | --- | --- | --- |
|  | **Total†**  **(N=3596)** | **Incident CVD**  **(N=102)** | **Men with incident CVD**  **(N=77)** | **Women with incident CVD**  **(N=25)** |
| **Baseline  WLC Score, mean ± SD** | 36.89±26.95 | 33.82±26.48 | 35.26±26.65 | 29.40±25.95 |
|  |  |  |  |  |
| **Baseline WLC, n (%)** |  |  |  |  |
| 0-19 | 1047 (29.1) | 35 (34.3) | 36 (33.8) | 9 (36.0) |
| 20-39 | 912 (25.4) | 24 (23.5) | 16 (20.8) | 8 (32.0) |
| 40-59 | 724 (20.1) | 20 (19.6) | 17 (22.1) | 3 (12.0) |
| 60-79 | 650 (18.1) | 18 (17.6) | 14 (18.2) | 4 (16.0) |
| 80-100 | 263 (7.3) | 5 (4.9) | 4 (5.2) | 1 (4.0) |

**†** Of the study sample considered at baseline (n=3698), information on incident CVD during the follow-up was missing for 102 study participants.
